# RNA Therapeutics in Cardiovascular Precision Medicine

**DOI:** 10.3389/fphys.2018.00953

**Published:** 2018-07-25

**Authors:** Ageliki Laina, Aikaterini Gatsiou, Georgios Georgiopoulos, Kimon Stamatelopoulos, Konstantinos Stellos

**Affiliations:** ^1^Department of Clinical Therapeutics, Alexandra Hospital, National and Kapodistrian University of Athens, Athens, Greece; ^2^Center of Molecular Medicine, Institute of Cardiovascular Regeneration, Goethe University Frankfurt, Frankfurt, Germany; ^3^Department of Cardiology, Center of Internal Medicine, Goethe University Frankfurt, Frankfurt, Germany; ^4^German Center of Cardiovascular Research, Rhein-Main Partner Site, Frankfurt, Germany; ^5^Cardiovascular Research Centre, Institute of Genetic Medicine, Newcastle University, Newcastle upon Tyne, United Kingdom; ^6^Department of Cardiology, Freeman Hospital, Newcastle upon Tyne Hospitals NHS Foundation Trust, Newcastle upon Tyne, United Kingdom

**Keywords:** cardiovascular precision medicine, RNA therapy, antisense oligonucleotides, ASO, silence interfering RNA, siRNA, aptamer, microRNA

## Abstract

Since our knowledge on structure and function of messenger RNA (mRNA) has expanded from merely being an intermediate molecule between DNA and proteins to the notion that RNA is a dynamic gene regulator that can be modified and edited, RNA has become a focus of interest into developing novel therapeutic schemes. Therapeutic modulation of RNA molecules by DNA- and RNA-based therapies has broadened the scope of therapeutic targets in infectious diseases, cancer, neurodegenerative diseases and most recently in cardiovascular diseases as well. Currently, antisense oligonucleotides (ASO), small interfering RNAs (siRNAs), and microRNAs are the most widely applied therapeutic strategies to target RNA molecules and regulate gene expression and protein production. However, a number of barriers have to be overcome including instability, inadequate binding affinity and delivery to the tissues, immunogenicity, and off-target toxicity in order for these agents to evolve into efficient drugs. As cardiovascular diseases remain the leading cause of mortality worldwide, a large number of clinical trials are under development investigating the safety and efficacy of RNA therapeutics in clinical conditions such as familial hypercholesterolemia, diabetes mellitus, hypertriglyceridemia, cardiac amyloidosis, and atrial fibrillation. In this review, we summarize the clinical trials of RNA-targeting therapies in cardiovascular disease and critically discuss the advances, the outcomes, the limitations and the future directions of RNA therapeutics in precision transcriptomic medicine.

## Introduction

Cardiovascular disease (CVD) is the leading cause of death and disability in developed countries despite advances in risk stratification strategies and treatment (Benjamin et al., 2017). Thus, the need for developing novel therapeutic strategies remains a major challenge in cardiovascular medicine. Several lines of evidence have expanded our understanding of RNA function beyond its role as an intermediate molecule between DNA and proteins to a dynamic and versatile regulator of gene expression (Kapranov et al., 2007; Mercer et al., 2009). Today, we know that RNA is edited (Stellos et al., 2016), modified (Stellos, 2017b), forms secondary and tertiary structures (Cate, 2016) and undergoes a tight, dynamic and in some cases reversible post-transcriptional regulation by a plethora of RNA-binding proteins (Cate, 2016; Stellos et al., 2016; Stellos, 2017b). To this end, RNA-targeting therapies are currently under clinical development by biotechnology companies expanding the range of “drug-able” targets. Small interfering RNAs (siRNAs) and microRNAs (miRNAs), the endogenous regulators of gene silencing, have been investigated as potential therapeutic agents. Synthetic siRNAs are used to inhibit the expression of the mRNA target, while miRNA-based therapeutics comprise miRNA inhibitors and miRNA mimics that antagonize and mimic the function of an endogenous miRNA, respectively. Synthetic antisense oligonucleotides and aptamers, a new class of either short DNA or RNA oligonucleotides, are also used to target the RNA. To date, RNA-targeting therapies are already being applied in various diseases including cancer (Moreno and Pego, 2014), infectious (Schluep et al., 2017), and neurodegenerative diseases (Scoles et al., 2017) as well. Nevertheless, RNA-targeting therapeutic modalities merit various chemical modifications prior to achieving greater stability and specificity, improved potency, and decreased toxicity (Kole et al., 2012; Wittrup and Lieberman, 2015).

The therapeutic potential of RNA-targeting therapies in the context of cardiovascular disease therapeutics is currently explored in multiple clinical trials. This review focuses on two approaches used to therapeutically target RNA, that is siRNA and ASOs, and summarizes the clinical trials of RNA-targeting therapies in cardiovascular diseases. Further, we critically discuss the advances, the outcomes, the limitations and the future directions of RNA therapeutics in precision transcriptomic medicine.

## Mechanisms of action and chemical modifications of DNA- and RNA-based therapies targeting RNA molecules

Therapeutic targeting of RNA (“transcriptomic medicine”) is currently based on two main approaches: single-stranded antisense oligonucleotides (ASO) and double-stranded RNA-mediated interference (RNAi). Below, we discuss the mechanisms of action of RNA-targeting therapies and chemical modifications introduced to improve drug design.

### Antisense oligonucleotides (ASOs)

**Figure 1:**
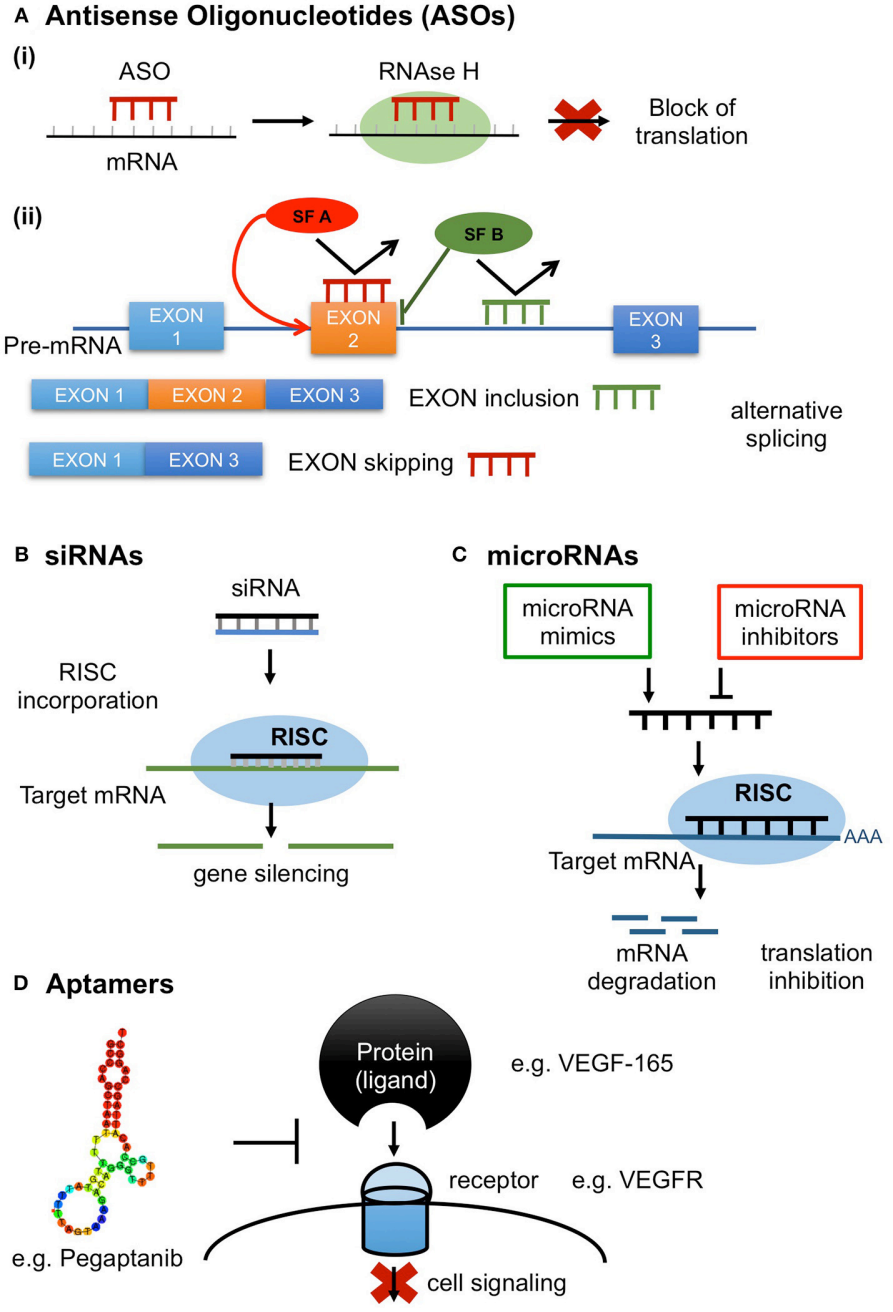
RNA therapeutics in action. **(A)** Antisense oligonucleotides (ASOs); short, synthetic, single-stranded oligodeoxynucleotides that modify protein expression through the following mechanisms **(Ai)** Inhibition of protein production by antisense gapmers through activation of the ribonuclease RNAase H resulting in target mRNA degradation; **(Aii)** Control of splicing by ASOs in alternative splicing. ASOs can modulate alternative splicing by preventing the binding of splicing factors (SF) resulting in translational arrest through ribosome attachment blocking; **(B)** siRNAs. Double stranded (ds) RNA is processed by Dicer, a dsRNA-specific ribonuclease, into 21–25 nucleotide-long ds siRNAs with 2 nucleotides in their 3′ overhang and 5′ phosphate groups. siRNAs are then recognized by the Argonaute 2 (AGO2) and loaded into the RNA-induced silencing complex (RISC) and unwind into their single strand components. AGO2, which is a component of RISC, cleaves the sense strand of siRNA and the antisense strand binds with perfect complementarity to the target mRNA resulting in target mRNA cleavage; **(C)** microRNAs. Induction or inhibition of gene expression by microRNA mimics or inhibitors. **(D)** Aptamers. Aptamers are single-stranded DNA or RNA molecules selected through a large oligonucleotide library, called SELEX, to bind a specific target with high selectivity and specificity. Common targets include small metal ion and organic molecules, proteins, viruses, bacteria and whole cells. Target recognition and binding involve three dimensional, shape-dependent interactions as well as hydrophobic interactions. Here is a schematic illustration of the aptamer Pegaptanib inhibiting the action of the target protein VEGF-165 by binding to its receptor VEGFR.

Antisense oligonucleotides comprise a promising class of synthetic agents designed to modulate gene expression (Shen and Corey, 2018). They are short, typically 20 base pairs (bp) in length, single-stranded DNA based oligonucleotides which inhibit protein translation by binding to the target mRNA in a sequence-specific manner via Watson-Crick base-pairing (Shen and Corey, 2018). Antisense oligonucleotides target various classes of nucleic acids inside the cell (pre-mRNA, mRNA, non-coding RNA). ASOs have been developed to exert various mechanisms of action depending on the location of hybridization and ASOs' chemical properties (Chan et al., 2006). ASOs inhibit protein production mainly through stimulation of RNAase H activity, which in turn results in target mRNA degradation (ASO “Gapmers”) (Crooke, 1999). ASOs can also induce alternative splicing by preventing binding of splicing factors (Dominski and Kole, 1993; Havens and Hastings, 2016), resulting in translational arrest through ribosome attachment blocking (steric hindrance) (Crooke, 1999; Figure 1).

Antisense oligonucleotides are subject to chemical modifications that can be utilized to improve their pharmacodynamic and pharmacokinetic properties (Crooke et al., 2017). Obstacles that have to be overcome are: (i) instability and degradation by nucleases, (ii) low cellular uptake and poor delivery to the tissues, (iii) inadequate binding affinity to target mRNA, and (iv) off-target effects and toxicity (Kole et al., 2012). To this purpose, phosphorothioate (PS) linkages between the nucleosides that form the backbone were introduced in replacement to phosphodiester bond generating the first generation ASOs (Crooke et al., 2017). Phoshodiester linkages are hydrophilic and highly charged, thus vulnerable to rapid degradation by nucleases. In contrast, PS linkages confer increased stability against nucleases and improve serum protein binding, thus facilitating tissue distribution and increasing ASOs pharmacokinetic profile (Dowdy, 2017). Sub-optimal affinity of the target mRNA leading to low potency was addressed by second and third generation ASOs, where 2′-O-Methyl (2′-OMe), 2′-O-methoxyethyl (2′-OMOE), and Locked Nucleic Acids (LNAs) are the leading types of chemical modifications, respectively (Wahlestedt et al., 2000). Collectively, chemical modifications result in improved stability and selective binding, thus enabling efficient delivery (Freier and Altmann, 1997).

### Small interfering RNAs (siRNAs)

RNA interference (RNAi) is a highly conserved natural process present in most eukaryotic cells in which double-stranded (ds) RNA molecules silence the post-transcriptional expression of specific genes (Siomi and Siomi, 2009). Small interfering RNAs (siRNAs) and microRNAs are small non-coding RNAs consisting major mediators of the RNAi process. siRNAs have been used as synthetic mediators of RNAi specifically designed to silence the expression of target genes (Siomi and Siomi, 2009). Unlike ASOs which are single-stranded oligodeoxynucleotides, siRNAs are double-stranded RNA molecules ranging in length from 19 to 25 bp. After they are exogenously transfected into the cell, either in a short form or in the form of long dsRNA molecules, they are further incorporated into the RNAi machinery (Siomi and Siomi, 2009). Long dsRNAs, transfected in low concentrations to prevent immune response through activation of the interferon pathway are cleaved by Dicer, a dsRNA-specific ribonuclease, into 21–25 nucleotide-long double-strand siRNAs with 2 nucleotides in their 3′ overhang and 5′ phosphate groups. siRNAs are then recognized by the Argonaute 2 (AGO2) and RNA-induced silencing complex (RISC) and unwind into their single strand components (Sledz et al., 2003; Liu et al., 2004; Meister, 2013). The sense strand is degraded and its complement (antisense) strand binds with perfect complementarity to the target mRNA sequence which is cleaved by AGO2 and degraded by exonucleases (Rand et al., 2005; Ozcan et al., 2015; Figure 1).

Specificity and off-target effects depend on the complementarity between an siRNA and the target gene (Jackson and Linsley, 2010). In fact, the off-target effect along with efficacy, delivery issues, immune system activation, and toxicity are challenges in siRNA therapeutic approach that have hampered their development into drug agents. Despite the fact that siRNAs are designed to silence specific targets, they could also knock down unintended genes, either through imperfect complementarity to non-targeted mRNAs or by entering the endogenous miRNA machinery (Jackson and Linsley, 2010). Poor delivery of siRNAs due to rapid degradation by circulating nucleases or rapid renal excretion has been addressed by introducing chemical modifications including PS modifications, hydrophobic ligands and encapsulation in nano carriers, in order to increase both protection and half-life enabling systemic delivery (Sledz et al., 2003; Kaczmarek et al., 2017). Chemical modifications have also been employed to inhibit innate immune system activation and release of pro-inflammatory cytokines induced by siRNAs through toll-like receptor signaling pathways (Shen and Corey, 2018).

### microRNAs (miRNAs)

MicroRNAs are endogenous small non-coding RNAs which similarly to siRNAs regulate gene expression at a post-transcriptional level, with the exception that are capable of silencing multiple mRNAs and not one target like siRNAs (Lam et al., 2015). miRNA maturation is a stepwise process during which miRNA is first transcribed as primary miRNA (pri-miRNA), which in turn is processed to a loop-structured precursor miRNA (pre-miRNA) of ~60- to 70 nucleotides (nt) by Drosha enzyme. Dicer, another RNase, cleaves the pre-miRNA yielding a miRNA duplex of ~22-nt which forms with the RISC a complex called miRISC. The complex is then unwinding and the sense strand is discarded leaving the single-stranded miRNA to bind to the mRNA target through partial complementary base pairing, resulting in gene silencing through translation suppression (Figure 1). Oligonucleotides that target miRNAs are known as antagomiRs, which bind with high complementarity to miRISC preventing the binding of the complex to the mRNA target. Oligonucleotides can also be synthezised to mimic miRNA function, providing another strategy for drug development (Li and Rana, 2014). ASOs targeting microRNA undergo chemical modifications in order to improve their pharmacokinetic profile, binding and resistance from nuclease cleavage (Rupaimoole and Slack, 2017). To date only one miRNA therapeutic agent, miravirsen which is an LNA-modified DNA phosphorothioate ASO that inhibits miR-122, has been evaluated in a clinical trial (NCT01200420) in patients with chronic hepatitis C, showing prolonged dose-dependent decreased HCV RNA levels without serious adverse events (SAE) (Janssen et al., 2013). Several experimental studies are investigating the use of miRNAs as therapeutic targets in atherosclerosis, coronary artery disease and myocardial infarction and clinical trials in humans are expected to test microRNAs either as antagomiRs or microRNA mimics in cardiovascular disease (Obad et al., 2011; Rayner et al., 2011; Bernardo et al., 2012).

### Aptamers

Aptamers are a new class of agents used both for diagnostic and therapeutic purposes. They are synthetic single-stranded DNA or RNA molecules selected for binding to a specific target through an iterative process called SELEX (systematic evolution of ligands by exponential enrichment) (Zhou and Rossi, 2017). Numerous aptamers have been generated to target a wide range of molecules, including small metal ion and organic molecules, proteins, viruses, bacteria and whole cells. The ability to form three dimensional interactions with their targets renders them comparable to monoclonal antibodies. In fact, they are often termed as “chemical antibodies” as they share functional capacities with monoclonal antibodies. On the other hand, aptamers have smaller size, higher affinity and selectivity as compared to monoclonal antibodies and importantly they possess a more flexible structure being able to bind to inaccessible targets for larger antibodies. Taken together, given these advantages as well as that aptamers can be synthesized *in vitro* and lack immunogenicity, these agents consist an attractive alternative therapeutic strategy to monoclonal antibodies (Zhou and Rossi, 2017). Moreover, besides binding their cognate protein, aptamers also act as antagonists efficiently inhibiting the function of a specific target. The first aptamer approved by the FDA, Macugen, is a typical aptamer antagonist (Figure 1). Macugen was designed to target vascular endothelial growth factor (VEGF) for the treatment of age-related macular degeneration (AMD), however did not show superior therapeutic effect than VEGF-specific monoclonal antibodies (Ng et al., 2006; Mousa and Mousa, 2010; Ferrara and Adamis, 2016).

## Clinically applicable targets in cardiovascular disease

**Apolipoprotein B** is an essential structural component of all atherogenic lipoproteins, including low-density lipoprotein cholesterol (LDL-C), responsible for LDL-C transport and removal and a major determinant of cardiovascular risk (Crooke and Geary, 2013). **Mipomersen** is a 20 nucleotide-long antisense oligonucleotide targeting apolipoprotein B (ApoB) mRNA indicated in homozygous familial hypercholesterolemia (HoFH) exerting its action by binding to ApoB mRNA and inhibiting the subsequent synthesis of the protein through RNase H activation.

The approval of mipomersen [Kynamro, Kastle Therapeutics] by FDA has paved the way towards investigating other possible targets using antisense technology in the field of CVD. One of the most promising targets is **proprotein convertase subtilisin/kexin-9 (PCSK9)**, which is responsible for reduced LDL receptor expression and uptake of LDL-C and transport into hepatocytes, resulting in elevated LDL-C circulating levels. Inclisiran is a long-acting RNAi therapeutic agent that inhibits the protein synthesis of PCSK9 and has been evaluated in a phase 2 randomized clinical trial showing promising results (Ray et al., 2017; **Table 2**). **Apolipoprotein C3 (APOC-III)** is another target currently being evaluated in patients with familial chylomicronemia syndrome. APOC-III exerts its atherogenic action by attenuating lipolysis of triglyceride-rich lipoproteins through lipoprotein lipase (LPL) inhibition that results in increased circulating levels of very low density lipoproteins and chylomicrons (Huff and Hegele, 2013). Increased levels of APOC-III are found in patients with hypertriglyceridemia and have been causally associated with metabolic syndrome and insulin resistance (Baldi et al., 2013). On the contrary, carriers of mutations disrupting APOC-III function presented 40% lower risk for coronary heart disease compared to non-carriers (TG and HDL Working Group of the Exome Sequencing Project et al., 2014). To date, Volanesorsen is a second generation antisense oligonucleotide that has been designed to target *APOC-III* mRNA and is currently being evaluated in phase 3 clinical trials in patients with familial chylomicronemia syndrome (The APPROACH Study, The COMPASS Study) (**Table 4**).

**Apolipoprotein(a)** is an essential component of lipoprotein(a) which has been identified as an independent risk factor for cardiovascular disease and calcific aortic valve stenosis (Danesh et al., 2000; Capoulade et al., 2015). The atherogenic properties of Lp(a) are attributed to the LDL-like particle and apo(a) components and to the latter's homology to plasminogen, as well as to its content of pro-inflammatory oxidized phospholipids (OxPL) (Wiesner et al., 2013).

Genome-wide association studies have identified **angiopoietin-like 3 (ANGPT3) and angiopoetin-like 4 (ANGPT4)** as potential cardiometabolic therapeutic targets to reduce cardiovascular burden. Several lines of evidence support a beneficial metabolic profile in patients with loss-of-function genetic variants in the gene encoding ANGPTL3 and ANGPTL4 expressed as low levels of plasma LDL cholesterol, high-density lipoprotein (HDL) cholesterol, triglycerides, and reduced insulin resistance (Robciuc et al., 2013; Dewey et al., 2016). An ASO to ANGPTL3 has been developed and recently investigated in human volunteers in a phase 1 clinical trial (Graham et al., 2017; Table 1).

**Table 1:** Phase 1 randomized clinical trials of RNA therapeutics in cardiovascular disease.

**Study registration number (ref)**	**Condition**	**Intervention arm**	**Comparator arm**	**Primary endpoint**	**Outcomes: % change from baseline (95% CI)**		**Adverse events**
					**Placebo**	**Study drug**	
**APOC-III mRNA**							
(Graham et al., 2013)	Healthy volunteers	Volanesorsen ISIS 304801 200 mg/weekly (*n* = 3) ^**^doses 50, 100 and 400 mg/weekly tested	Placebo (*n* = 4)	% change from baseline in apoC-III and TG 1 week post- treatment	ApoC-III: −11^*^ TG:28.5^*^	ApoC-III: −70.5^*^ TG: −43.1^*^	**Injection site reaction** Plc: 0% Volanesorsen: 52% **Elevated CRP** Plc: not reported Volanosersen: 28%
**PCSK9 mRNA**							
(Fitzgerald et al., 2014) NCT01437059	Healthy adults with LDL>115 mg/dl	ALN-PCS 0.4 mg/kg single dose (*n* = 6) ^**^doses 0.015, 0.045, 0.09, 0.150, 0.250 mg/kg tested (*n* = 24)	Placebo (*n* = 8)	Safety and tolerability	PCSK9: −8.7^*^ LDL-C: −24^*^	PCSK9: −58.6^*^^,^^†^ LDL-C: −36.1^*^^,^^†^	**Rash** Plc: 50% ALN-PCS: 50% **Headache** Plc:25% ALN-PCS: 20.8%
(Fitzgerald et al., 2016) NCT02314442	Healthy adults with LDL>100 mg/dl TG < 400 mg/dl or statin therapy	ALN-PCS 300 mg/month (*n* = 12) ^**^doses 125, 250, 500 mg at single or multiple doses infusion tested	Placebo (*n* = 17)	Safety, pharmacokinetics and lipid parameters 4 weeks post- treatment	PCSK9: −0.6 (−24.2 to 30.4) (sd, *n* = 6) LDL-C: −10.9 (−26.0 to 7.1) (sd, *n* = 6) PCSK9: 16.9 (−2.4 to 40.0) (dd, *n* = 11) LDL-C: −14.2 (−30.2 to 5.5) (dd, *n* = 11)	PCSK9: −74.5 (−82.1 to −63.6) (sd, *n* = 3) LDL-C: −50.0 (−60.7 to −36.3) (sd, *n* = 3) PCSK9: −71.8 (−77.4 to −64.8) (dd no statin, *n* = 6) LDL-C: −59.7 (−68.7 to −48.1) (dd no statin, *n* = 6) PCSK9: −79.9 (−85.4 to −72.5) (dd with statin, *n* = 6) LDL-C: −45.1 (−61.6 to −21.4) (dd with statin, *n* = 6)	**Cough, Musculoskeletal pain, Nasopharyngitis** Plc: 0 ALN-PCS: 2 (11.1%) **Headache** Plc: 2 (16.7%) ALN-PCS: 6 (18.2%) **Backpain** Plc: 2 (16.7%) ALN-PCS: 5 (15.2%) **Diarrhea** Plc: 3 (25.0%) ALN-PCS:4 (12.1%) **Nasopharyngitis** Plc: 1 (8.3%) ALN-PCS: 5 (15.2%)
(van Poelgeest et al., 2015) NCT01350960	Healthy adults with LDL-C>100 mg/dl	SPC5001 siRNA 5 mg/kg (*n* = 6) ^**^doses 0.5 and 1.5 mg/kg tested	Placebo (*n* = 6)	Safety, pharmacokinetics and lipid parameters 2 (PSCK9) or 3 weeks (LDL-C) post-treatment	PCSK9: −49.0 (−58.5, −37.2)^¥^ LDL-C: −0.36 (−0.75 to 0.03)^¥^		**Injection site reaction** Plc: not reported SPC5001:44% **Headache** Plc: 33% SPC5001: 61% **Tiredness** Plc: 17% SPC5001: 56% Renal tubular toxicity (4/6 highest dose)
**APO(a) mRNA**							
(Tsimikas et al., 2015) 2012-004909-27	Healthy adults With Lp(a)>100 mg/L	ISIS-APO(a)Rx 200 mg (*n* = 12) ^**^doses 50, 100, 300, and 400 mg at single or multiple doses tested	Placebo (*n* = 4) (sd) (*n* = 6) (md)	% change in Lp(a) 4 weeks (single) or 12(md) post treatment	Lp(a): ^#^ (single dose) Lp(a): −59^*^, ^†^, ^¥^ (multiple doses)		2 discontinuations due to injection site adverse event and flu-like syndrome
(Viney et al., 2016) NCT02414594	Healthy adults with Lp(a)>75 nmol/L	APO(a)-LRx 120 mg single dose (*n* = 6) and 40 mg (*n* = 8) for multiple doses ^**^doses 10, 20, 40 and 80 mg for single dose and 10 and 20 mg at multiple doses tested	(*n* = 13)	%change in Lp(a), safety and tolerability One (single dose) or two (multiple doses) weeks post-treatment	Lp(a): −84.5 (−112.6 to −65.2) (single dose)^¥^ Lp(a): − 82·4% (−99.8 to −67.6) (multiple dose)^¥^		No SAE, local injection-site reactions, influenza-like symptoms, or other safety issues
**ANGPTL3 mRNA**							
(Graham et al., 2017) NCT02709850	Healthy adults with LDL-C>70 mg/dl and TG>90 mg/dl	ANGPTL3-LRx 80 mg (single dose) (*n* = 3) and 60 mg (multiple doses) (*n* = 6) ^**^doses 10, 20 and 40 mg at single or multiple doses tested	Placebo (*n* = 4) (single dose) (*n* = 8) (multi doses)	Safety, pharmacokinetics pharmacodynamics 1 week post- treatment	ANGPTL3: 0.3 ± 17.4^*^ LDL-C: −0.3 ± 24.5^*^ (single dose) ANGPTL3: −1.6± 15.4^*^ LDL-C: 13.6 ± 12.1^*^ (multiple doses)	ANGPTL3: −61.7 ± 1.1^*^ LDL-C: −24.0 ± 5.9^*^ (single dose) ANGPTL3: −84.5± 5.1^*^^,^^†^ LDL-C: −32.9± 10.4^*^^,^^†^ (multiple doses)	**Headache** Plc: 1 ANGPTL3-LRx:2 **Dizziness** Plc: 2 ANGPTL3-LRx: 1 No SAE

*LDL-C, low-density lipoprotein cholesterol; apoB, apolipoprotein B; TC, total cholesterol; Lp(a), lipoprotein a; CI, confidence intervals; PCSK9, proprotein convertase subtilisin/kexin type 9; ANGPTL3, angiopoietin-like 3; sd, single dose; dd, double dose; md, multi dose; Plc, placebo; SAE, serious adverse events*.

* *95% CIs for % change are not provided. Standard deviation or interquartile range (25 to 75th percentile) are shown where available*.

** *Indicate different dosage scheme*.

† *Indicates observed statistical significance < 0.05*.

¥ *Indicates relative changes in treatment group as compared to placebo*.

# *No numeric estimates are provided*.

Coagulation factors are currently being investigated as potential therapeutic targets and anti-coagulant aptamers have been designed and tested in patients with coronary artery disease. **REG1** is a two-component system: RB006 is a single-stranded, nucleic acid aptamer and comprises the anticoagulant component inhibiting **IX factor** (Rusconi et al., 2002). Its action is reversed by the antidote component of the REG1, RB007 that binds and neutralizes RB006. Phase 2 clinical trials have evaluated the efficacy of REG1 in patients with stable CAD or ACS undergoing percutaneous coronary intervention (PCI) compared to unfractionate heparin, reporting a beneficial bleeding profile along with reduced thrombotic complications in patients receiving the aptamer nucleic acid agent (Cohen et al., 2010; Povsic et al., 2014; Table 2). However, a subsequent study comparing the efficacy between REG1 anticoagulation system and bivalirudin in patients undergoing PCI in terms of periprocedural ischemic complications and major bleeding was terminated due to severe allergic reactions reported in subjects receiving the RNA aptamer (Ganson et al., 2016; Lincoff et al., 2016). Of note, REG1 was not associated with reduced bleeding and ischemic events compared to bivalirudin (Lincoff et al., 2016). **ARC-1779** is a 39-nucleotide modified DNA aptamer designed to target **von Willebrand factor** (Gilbert et al., 2007) assessed in phase 2 studies in patients, undergoing carotid endarterectomy and patients with acute myocardial infarction undergoing PCI, respectively. However, both trials have been terminated (NCT00742612 and NCT00507338) due to slower enrolment than expected and unfeasible mode of drug administration, respectively. Similarly, **NU172** is a 26-nucleotide unmodified DNA aptamer targeting **thrombin**. Results from a phase 1 study provide initial evidence that this agent could achieve reversal of anticoagulation without the need of an antidote. An open-label phase 2 clinical trial has been conducted to evaluate the therapeutic efficacy of NU172 in patients undergoing CABG (NCT00808964). Its outcome is currently unknown.

**Table 2:** Phase 2 randomized clinical trials of RNA therapeutics in cardiovascular disease.

**Study registration number (ref)**	**Condition**	**Intervention arm**	**Comparator arm**	**Primary endpoint**	**Outcomes: % change from baseline (95% CI)**		

					**Placebo**		**Study drug**
**APOC-III mRNA**							
(Gaudet et al., 2015) NCT01529424	Hypertriglyceridemia (>350 mg/dl or >225 mg/dl added to fibrate)	Volanosersen (ISIS 304801) 300 mg/weekly (*n* = 21) ^**^ doses 100 and 200 mg/weekly have been tested	Placebo (*n* = 24)	% change in apoC-III levels from baseline to end of treatment	ApoC-III: 4.2 ± 41.7^*^ TG: 20.1 ± 72.0^*^ ApoC-III: 2.2 ± 25.2^*^ TG: −7.7 ± 33.8^*^		ApoC-III: 79.6 ± 9.3^*^^,^^†^ (volanosersen only, *n* = 11) TG: −70.9 ± 14.1^*^^,^^†^ ApoC-III: 70.9 ± 13.0^*^^,^^†^ (+fibrate, *n* = 10) TG: −64.0 ± 8.9^*^^,^^†^
(Digenio et al., 2016) NCT01647308	Type 2 DM and Hypertriglyceridemia	Volanesorsen 300 mg/weekly (*n* = 10)	Placebo (*n* = 5)	% change in apoC-III from baseline to end of treatment	ApoC-III:−7.3 ± 14^*^ TG:−9.9 ± 19.9^*^ LDL-C: −5.5 ± 7.2^*^ ApoB: −10.4 ± 8.5^*^ HbA1c: −0.78 ± 0.71^*^		ApoC-III:−87.5 ± 5.4^*^^,^^†^ TG: −69.1 ± 10.1^*^^,^^†^ LDL-C: 0 ± 26.3^*^ ApoB: −20.8 ± 15.9^*^ HbA1c: −0.44 ± 0.39^*^^,^^†^
(Yang X. et al., 2016)	FCS Hypertriglyceridemia	Volanosersen 300 mg/weekly (*n* = 24) ^**^doses 100 and 200 mg/weekly tested	Placebo (*n* = 24)	% changes in apoCIII-apoB, apoCIII-apoAI, and apoCIII-Lp(a)	apoCIII-apoB: −82.3 ± 11.7^*^^,^^†^^,^^¥^ apoCIII- Lp(a): −81.3 ± 15.7^*^^,^^†^^,^^¥^ apoCIII-apoA1: −80.8 ± 13.6^*^^,^^†^^,^^¥^ (baseline to end of treatment)		
**PCSK9 mRNA**							
(Ray et al., 2017) ORION-1 NCT02597127	Hypelipidemia	Inclisiran 300 mg per quarterly (*n* = 119) ^**^ doses 200 and 500 mg, single or double quarterly dose tested	Placebo (*n* = 125)	% change from baseline in LDL cholesterol 3 months post-treatment	LDL-C: 2.1 (−2.9 to 7.2) (*n* = 64, single dose) PCSK9: 2.2 ± 23.4^*^ (*n* = 64, single dose) LDL-C: 1.8 (−2.6 to 6.3) (*n* = 61, double dosage) PCSK9: −1.2 ± 20.7^*^ (*n* = 61, double dose)		LDL-C: −38.4 (−43.6 to −33.2) (*n* = 60, single dose) PCSK9: −56.0 ± 19.2^*^^,^^†^ (*n* = 60, single dose) LDL-C: −52.6 (−57.1. to −48.1) (*n* = 59, double dose) PCSK9: −69.1 ± 12.1^*^^,^^†^ (*n* = 59, double dose)
**APO(a) mRNA**							
(Viney et al., 2016) NCT02160899	Healthy adults with Lp(a)>125 nmol/L (cohort A) or Lp(a)>437 nmol/L (cohortB)	ISIS-APO(a)Rx 100–300 mg Cohort A(*n* = 24) Cohort B (*n* = 8)	Placebo (*n* = 29)	%change in Lp(a), safety and tolerability end of or 2 weeks post-treatment	Lp(a): −62.8 (−71.9 to −53.8) (cohort A)^¥^ Lp(a): −67.7% (−80.8 to −54.5) (cohort B) ^¥^		
**IX FACTOR**							
(Povsic et al., 2014) (RADAR-PCI) NCT00932100	ACS patients undergoing PCI	Pegnivacogin 1 mg/kg with 25%, 50%, 75%, or 100% anivamersen reversal (*n* = 277)	UFH (*n* = 111)	$Composite ischemic endpoint and bleeding through 30 days	Bleeding: 7% (100% reversal) vs. 11% Ischemic events: 4.4 vs. 7.3% TVR: 1.1 vs. 0.9% MI: 4 vs. 6.4% Angiographic complications: 11.2 vs. 10.8%		
**CHEMOKINE C-C MOTIF-LIGAND 2**							
(Menne et al., 2017)	Type 2 DM	Emapticap (*n* = 50)	Placebo (*n* = 25)	Change in urinary ACR at the end of treatment	−15%^*^		−29%^*^^,^^†^

*FH, familial hypercholesterolemia; CHD, coronary heart disease; CVD, cardiovascular disease; HCL, hypercholesterolemia; LDL-C, low-density lipoprotein cholesterol; apoB, apolipoprotein B; TC, total cholesterol; Lp(a), lipoprotein a; CI, confidence intervals; PCSK9, proprotein convertase subtilisin/kexin type 9; FCS, familial chylomicronemia syndrome; ACS, acute coronary syndrome; PCI, percutaneous coronary intervention; DM, diabetes mellitus; TVR, target vessel revascularization; MI, myocardial infarction; ACR, albumin/creatinine ratio*.

* *95% CIs for % change are not provided. Standard deviation or interquartile range (25 to 75th percentile) are shown where available*.

** *Indicate different dosage scheme*.

† *Indicates observed statistical significance < 0.05*.

¥ *Indicates relative changes in treatment group as compared to placebo*.

*$Death, non-fatal MI, urgent TVR, or recurrent ischemia*.

**Chemokine C-C motif-ligand 2** (CCL2) is a pro-inflammatory cytokine involved in the development of insulin resistance and macrophage infiltration and recent evidence supports a role of CCL2 in diabetic nephropathy (Carr et al., 1994). A CCL2 antagonizing L-RNA aptamer (Spiegelmer) was found to improve renal function in experimental studies and after confirming a safety profile in a phase 1 clinical trial in humans, emapticappegol (NOX-E36) was evaluated in a phase 2a study in patients with type 2 diabetes mellitus and albuminuria (Table 2). Patients treated with emapticappegol presented a trend toward reduced urinary albumin excretion and HbA1c, suggesting a promising role of this CCL2 inhibitor in both kidney disease and diabetes mellitus (Menne et al., 2017).

Elevated levels of **C-reactive protein** (CRP) are associated with high cardiovascular risk (Strandberg and Tilvis, 2000) and could consist a potential target in RNA precision medicine. ISIS-CRPRx is an ASO complementary to the coding region of the human CRP mRNA and in a phase 1 double-blind placebo-controlled study was administered in healthy volunteers during the acute-phase response to endotoxin challenge (Noveck et al., 2014). Pre-treatment with ISIS-CRPRx attenuated the expected endotoxin induced increase in CRP levels in a dose-dependent manner. The antisense agent was well-tolerated in all doses tested (Noveck et al., 2014). Subsequently, this second generation ASO was assessed in a phase 2 clinical trial in patients with paroxysmal atrial fibrillation and an implanted dual chamber permanent pacemaker. The rationale of the clinical trial was based on the association between atrial fibrillation (AF) and inflammation and evidence supporting increased CRP levels as a risk for AF development and perpetuation (Dernellis and Panaretou, 2004; Marcus et al., 2010; Liu et al., 2011; Pena et al., 2012). However, no reduction in AF burden was observed in patients treated with ISIS-CRPRx despite substantial decrease in CRP levels (Sugihara et al., 2015). The potential anti-inflammatory effect of ISIS-CRPRx has also been explored in rheumatoid arthritis in a phase 2 clinical trial showing a dose-dependent reduction of high sensitivity CPR (hs-CRP) at 36 days. In specific, the group receiving 400 mg ISI-CRPRx demonstrated a decrease equal to 76.7% in hs-CRP compared with a 14.4% decrease in the placebo group at 36 days which was lost by day 92 (Warren et al., 2015).

Small interfering RNAs and ASOs have been developed for the treatment of **transthyretin** (TTR) amyloidosis, a progressive heart disease causing severe congestive heart failure. After siRNAs encapsulated in lipid nanoparticles were shown to successfully induce transthyretin knockdown in patients with TTR amyloidosis in a phase 1 study (Coelho et al., 2013), a subsequent phase 2 study evaluated patisiran as a potential therapeutic strategy in TTR mediated familial amyloidotic polyneuropathy (Suhr et al., 2015). The ENDEAVOUR study was a phase 3 double-blind placebo-controlled clinical trial that evaluated the safety and efficacy of revusiran in patients with TTR mediated familial amyloidotic cardiomyopathy but was withdrawn due to safety concerns (NCT02319005) (Table 4). Furthermore, a specific TTR antisense oligonucleotide (IONIS-TTR) was evaluated in an open label study examining functional and structural cardiac parameters in patients with either hereditary or wild type TTR amyloidosis. Overall, the ASO was well-tolerated slowing down the progression of disease, as expressed by reduced left ventricular wall thickness and left ventricular mass, improved global systolic strain, 6-min walk test and NYHA class (Benson et al., 2017).

## RNA-targeting therapeutics in randomized clinical trials

### Apolipoprotein B

#### Mipomersen

The efficacy of subcutaneous administration of mipomersen at 200 mg/weekly dosage has been explored in phase 3 clinical trials. In particular, Raal et al., reported a mean percentage reduction in LDL-C levels, of ~25% (95% CI −31.6 to −17.7) in patients older than 12 years old with homozygous FH already receiving the maximum tolerated dose of a lipid-lowering drug, compared with a ~3% decrease in the placebo group. Twenty-six out of thirty-four treated patients experienced injection-site reactions, and four presented a significant increase in alanine aminotransferase (Raal et al., 2010; Table 3).

**Table 3:** Phase 3 randomized clinical trials of RNA therapeutics in cardiovascular disease.

**Study registration number (ref)**	**Phase**	**Condition**	**Intervention arm**	**Comparator arm**	**Primary endpoint**	**Outcomes: % change from baseline (95% CI)**	
						**Placebo**	**Study drug**
**APOB mRNA**							
(Raal et al., 2010) NCT00607373	3	Homozygous FH	Mipomersen 200 mg/weekly (*n* = 34)	Placebo (*n* = 17)	% change in LDL-C 2 weeks post-treatment	LDL-C: −3.3 (−12.1 to 5.5) apoB: −2.5 (−9.0 to 3.9) TC: −2.0 (−9.6 to 5.6) Lp(a): −7.9 (−19.1 to 3.4)	LDL-C: −24.7 (−31.6 to −17.7) apoB: −26.8 (−32.7 to −20.8) TC: −21.2 (−27.4 to −15.0) Lp(a):−31.1 (−39.1 to −23.1)
(McGowan et al., 2012) NCT00794664	3	Heterozygous FH ± CHD	Mipomersen 200 mg/weekly (*n* = 39)	Placebo (*n* = 18)	% change in LDL-C 2 weeks post-treatment	LDL-C: 12.5 (−10.7 to 35.8) apoB: 11.4 (−6.9 to 29.7) TC: 11.2 (−6.2 to 28.5) Lp(a): −1.5 (−14.2 to 11.3)	LDL-C: −35.9 (−51.3 to −15.3) apoB: −35.9 (−43.3 to −28.4) TC: −28.3 (−34.9 to −21.7) Lp(a): −32.7 (−43.3 to −22.0)
(Stein et al., 2012) NCT00706849	3	Heterozygous FH + stable CAD	Mipomersen 200 mg/weekly (*n* = 83)	Placebo (*n* = 41)	% change in LDL-C 2 weeks post-treatment	LDL-C: 5.2 (−0.5 to 10.9) apoB: 7.02 (1.8 to12.2) TC: 3.85 (−0.2 to 7.9) Lp(a): 0.0 (−8.0 to 13.0)	LDL-C: −28.0 (−34.0 to −22.1) apoB: −26.3 (−31.2 to −21.4) TC: −19.4 (−23.7 to −15.2) Lp(a): −21.1 (−37.9 to 0.0)
(Visser et al., 2012) (ASSIST) (75) NCT00707746	3	High CVD risk Statin intolerance	Mipomersen 200 mg/weekly (*n* = 21)	Placebo (*n* = 12)	% reduction in LDL-C 2 weeks post-treatment	LDL-C: −2.0 ± 8.4^*^apoB: −4.3 ± 7.5^*^ TC: −1.8 ± 6.5^*^ Lp(a): 0.0 ± 8.6^*^	LDL-C: −47.3 ± 18.5,^*^^,^^†^ apoB: −46.2 ± 19.5^*^^,^^†^ TC: −36.9 ± 14.7^*^^,^^†^ Lp(a): −27.1 ± 31.2^*^^,^^†^
(Cromwell et al., 2012) (abstract)	3	HCL and High CHD risk	Mipomersen 200 mg/weekly (*n* = 105)	Placebo (*n* = 53)	% change in LDL-C 2 weeks post-treatment	LDL-C: −5% (−11, 2)	LDL-C: −37% (−42% to −32%) apoB: −38%^*^^,^^†^ TC: −26% Lp(a): −24% ^‡^
(Thomas et al., 2013) NCT00770146	3	HCL and High CHD risk ± CHD	Mipomersen 200 mg/weekly (*n* = 105)	Placebo (*n* = 52)	% reduction in LDL-C 2 weeks post-treatment	LDL-C: −4.5 ± 24.22^*^ apoB: −4.1 ± 18.09^*^ TC: −2.7 ± 14.58^*^ Lp(a): 0.0 (−16.0, 17.6)^*^	LDL-C: −36.9 ± 26.85^*^^,^^†^ apoB: −37.5 ± 23.59^*^^,^^†^ TC: −26.4 ± 18.65^*^^,^^†^ Lp(a): −25.6 (−40.0, −7.8)^*^^,^^†^

*FH, familial hypercholesterolemia; CHD, coronary heart disease; CAD, coronary artery disease; CVD, cardiovascular disease; HCL, hypercholesterolemia; LDL-C, low-density lipoprotein cholesterol; apoB, apolipoprotein B; TC, total cholesterol; Lp(a), lipoprotein a*.

* *95% CIs for % change are not provided. Standard deviation or interquartile range (25 to 75th percentile) are shown where available*.

† *Indicates observed statistical significance < 0.05*.

‡ *Relative changes from baseline are not provided. Baseline and post-treatment absolute values are shown*.

Similar results were reported by McGowan et al., who randomly assigned 58 patients with heterozygous FH and/or coronary heart disease (CHD) to either placebo or s.c. mipomersen 200 mg/weekly on top of the maximally tolerated dose of lipid-lowering drugs. The mipomersen group presented a 36% reduction of LDL-C from a baseline of 278 mg/dL in comparison to the placebo group which presented a 13% reduction from a baseline of 250 mg/dL. Conventional hypolipidemic drugs have limited effect on Lp(a). Surprisingly, in this study mipomersen induced a significant reduction in Lp(a) compared to placebo (−33 vs. −1.5%). Adverse events included injection site reactions, alanine transaminase increase and flu like symptoms (McGowan et al., 2012). Another study of similar design and population, that is patients with heterozygous FH and coronary artery disease, reported similar results concerning percentage change of LDL-C up to week 28. In specific, mipomersen decreased mean LDL-C by 28.0% compared with 5.2% increase with placebo. Moreover, mipomersen significantly reduced apolipoprotein B (−26.3%), total cholesterol (−19.4%), and Lp(a) (−21.1%) compared with placebo. No significant change occurred in HDL-C. Adverse events included injection site reactions and flu-like symptoms (Stein et al., 2012; Table 3).

The efficacy of mipomersen was also investigated in hypercholesterolemic subjects of high cardiovascular risk. In specific, 158 patients on maximally tolerated lipid lowering agents statin and LDL-C>100 mg/dL were randomized into receiving placebo (*n* = 53) or mipomersen (*n* = 105) for a 26-week period. Mean % change in LDL-C was −37% with mipomersen vs. −5% with placebo. No changes in HDL-C were observed, while on the contrary significant reductions were established in apoB (38%), total cholesterol (26%) and Lp(a) (24%) (Cromwell et al., 2012). Along this line, another study evaluated the efficacy of mipomersen in patients with baseline LDL cholesterol levels>100 mg/dL with or at high risk for CHD already receiving maximally tolerated lipid-lowering therapy. Mipomersen reduced LDL cholesterol by 36.9% compared to placebo's effect of 4.5%. Target LDL cholesterol < 100 mg/dl was achieved in 76% of mipomersen and 38% of placebo patients. Mipomersen conferred significant reductions in other lipid parameters as well. As in previous mipomersen studies, most common adverse events included injection site reactions (78% with mipomersen vs. 31% with placebo) and flu-like symptoms (34% with mipomersen vs. 21% with placebo) (Thomas et al., 2013; Table 3).

Finally, mipomersen was evaluated in high-risk patients with statin intolerance. LDL cholesterol decreased by 47.3%, with a parallel decrease in apoB by 46.2% and Lp(a) by 27.1%. Injection site reactions and flu-like symptoms were the most common adverse events resulting in 18% of the mipomersen-treated patients and 17% of the placebo-treated patients discontinuation of therapy. Among mipomersen treated patients, 33% of them presented liver function tests above three times the upper limit of normal (Visser et al., 2012; Table 3).

Interestingly, Duell et al. (2016) sought to assess the rate of major adverse cardiovascular events (MACE) across a follow-up period of 24 months in patients with FH having received mipomersen for at least 12 months in a *post-hoc* analysis of three RCTs and one open-label study (Santos et al., 2015). Patients after mipomersen initiation treatment experienced 13 MACE in comparison to 146 MACE identified in the 2-year period previous to the mipomersen therapy. In fact, the authors report that FH patients after mipomersen treatment initiation present 94.7% lower odds of experiencing MACE compared with the pre-treatment period.

### Apolipoprotein C-III

#### Volanesorsen

Volanesorsen is a second-generation 2′-O-methoxyethyl chimeric ASO that is designed specifically to reduce levels of *APOC-III* messenger RNA (mRNA). Through ribonuclease H1, volanesorsen induces the degradation of the target mRNA and inhibits the production of the APOC-III protein. This glycoprotein plays a regulative role on lipoprotein metabolism and single nucleotide polymorphisms (SNPs) in the APOC-III gene are emerging as a cause of severe hypertriglyceridemia. A recent meta-analysis found evidence that two SNPs in APOC-III are associated with increased CHD risk. In specific two polymorphisms, SstI and T-455C, increased the odds for CHD development by up to 48 and 77%, respectively (Li et al., 2016).

In a phase 1 study, healthy volunteers received either placebo (*n* = 8) or volanesorsen (ISIS 304801) (*n* = 25) and presented a deep dose-dependent reduction up to ~90% and up to ~80% of APOC-III and triglyceride levels, respectively 4 weeks post-treatment (Graham et al., 2013; Table 1). This led to a number of phase 2 clinical trials investigating the effect of APOC-III inhibition in subjects with hypertriglyceridemia.

Volanosersen was tested both as monotherapy and as an adjunct to fibrates in a placebo-controlled RCT. When administered as a single agent in fifty-seven subjects, volanosersen resulted in a dose-dependent and prolonged reduction in both plasma apoC-III (percentage decrease of 63.8 in the 200-mg group vs. an increase of 4.2% in the placebo group) and triglyceride levels (−57.7% in the 200-mg group vs. 20.1%). Similar results were observed when it was administered as an add-on treatment to fibrates (Digenio et al., 2016; Table 2). In addition, a phase 2 study explored the effect of volanesorsen in subjects with hypertriglyceridemia and poorly controlled type 2 diabetes. Volanosersen apart from significantly reducing apoC-III (−87.5% vs. −7.3%) and triglyceride levels (−69 vs. −9.9%) compared to placebo, also improved glycemic control, expressed as −0.44% reduction of HbA1c at the end of follow-up, and increased insulin sensitivity. Both findings were associated with suppression of apoC-III and triglyceride levels (Bennet et al., 2008; Table 2). Yang et al., using high-throughput ELISA to capture apoB, Lp(a) and apoA-I in plasma, identified significantly reduced apoC-III levels on these individual lipoproteins as apoCIII-apoB, apoCIII-Lp(a), and apoCIII- apoAI complexes in subjects who received volanosersen compared to those who received placebo (Yang X. et al., 2016; Table 2).

Ongoing studies with volanesorsen include the APPROACH [The APPROACH Study: A Study of Volanesorsen (Formerly ISIS-APOCIIIRx) in Patients with Familial Chylomicronemia Syndrome, NCT02211209] trial, the COMPASS [The COMPASS Study: A Study of Volanesorsen (Formally ISIS-APOCIIIRx) in Patients with Hypertriglyceridemia, NCT02300233] trial, and the BROADEN [The BROADEN Study: A Study of Volanesorsen (Formerly ISIS-APO-CIIIRx) in Patients with Partial Lipodystrophy, NCT02527343] trial (Table 4).

**Table 4:** Ongoing clinical trials on RNA therapeutics in cardiovascular disease.

**Trial registration number**	**Status**	**Study design**	**Phase**	**Condition (n)**	**Intervention arm**	**Comparator**	**Estimated completion date**
**PCSK9**							
NCT03060577 (ORION-3)	Active-not recruiting	Open label	2	FH (*n* = 490)	Inclisiran	Evolocumab	January 2022
NCT02963311 (ORION 2)	Recruiting	Open-label	2	Homozygous FH (*n* = 10)	ALN-PCS	SOC	December 2018
NCT03159416 (ORION-7)	Active, not recruiting	Open-label	1	HCL and renal impairment (*n* = 24)	Inclisiran		September 2018
**APOC-III**							
NCT02527343 (BROADEN Study)	Active, not recruiting	RDBPC	2/3	Familial Partial Lipodystrophy (*n* = 60)	Volanesorsen	Placebo	September 2021
NCT02900027	Recruiting	RDBPC	1	Hypertriglyceridemia (*n* = 56)	APOC-III-L-Rx	Placebo	September 2017
**ANGPT3**							
NCT03371355	Recruiting	RDBPC	2	Hypertriglyceridemia, Type 2 DM and NAFLD (*n* = 144)	ISIS 703802		May 2019
NCT03455777	Not yet recruiting	Open-Label	2	Homozygous FH (*n* = 3)	ISIS 703802		December 2018
NCT03360747	Recruiting	Open-Label	2	FCH (*n* = 3)	ISIS 703802		September 2018
**APO(a)**							
NCT03070782	Active, not recruiting	RDBPC	2	Hyperlipoproteinemia(a) and CVD (*n* = 270)	ISIS 681257	Placebo	November 2018
**GCGR**							
NCT02824003	Active, not recruiting	RDBPC	2	Type 2 DM (*n* = 15)	ISIS-GCGRRx	Placebo	May 2017
NCT02583919	Active, not recruiting	RDBPC	2	Type 2 DM (*n* = 80)	ISIS-GCGRRx	Placebo	March 2017
**Completed trials with unpublished data**							
**VEGF-A**							
NCT02935712	Completed	RSBPC	1	CVD (*n* = 44)	AZD8601	Placebo	January 2018
**APOC-III**							
NCT02211209 (APPROACH Study)	Completed	RDBPC	3	FCS (*n* = 67)	Volanosersen	Placebo	March 2017
NCT02300233 (COMPASS Study)	Completed	RDBPC	3	Hypertriglyceridemia (*n* = 114)	Volanosersen	Placebo	January 2017
**TTR**							
NCT02319005 (ENDEAVOR)	^*^Terminated	RDBPC	3	Cardiac Amyloidosis (*n* = 206)	ALN-TTRSC (revusiran)	Placebo	December 2017

* *Due to an imbalance of mortality in the revusiran arm as compared to placebo. RDBPC, randomized double-blind placebo controlled; RSBPC, randomized single-blind placebo controlled; PCSK9, proprotein convertase subtilisin/kexin type 9; FH, familial hypercholesterolemia; HCL, hypercholesterolemia; DM, diabetes mellitus; NFLD, non-alcoholic fatty liver disease; CVD, cardiovascular disease; Apo(a), apolipoprotein a; ApoC-III, apolipoprotein C-III; ANGPTL3, angiopoietin-like 3; GCGR, glucagon receptor; FCS, familial chylomicronemia syndrome; VEGF, vascular endothelial growth factor; TTR, transthyretin*.

### PCSK9

Treatment with PCSK9 inhibitors, evolocumab and alirocumab, reduce LDL-C levels by ~60% (Desai et al., 2017). Interestingly, the FOURIER (Further Cardiovascular Outcomes Research With PCSK9 Inhibition in Subjects With Elevated Risk) study has shown additional cardiovascular benefit exerted by PCSK9 inhibition besides lipid lowering effect. Evolocumab significantly reduced the risk of the composite endpoint of cardiovascular death, myocardial infarction, stroke, hospitalization for unstable angina, or coronary revascularization by 15% as compared to placebo matched patients (HR 0.85, 95% CI 0.79–0.92) (Sabatine et al., 2017). According to recently released results from meta-analysis of 35 randomized controlled trials PCK9 inhibition is not associated with improved all-cause (OR 0.71, 95% CI 0.47–1.09) or cardiovascular mortality (OR 1.01, 95% CI 0.85–1.19). However, a metaregression analysis revealed an association between higher baseline LDL-C and an all-cause mortality benefit (Karatasakis et al., 2017).

#### Inclisiran

Inclisiran (formerly known as ALN-PCS) is an investigational GalNAc-conjugated siRNA targeting PCSK9, designed for the treatment of hypercholesterolemia. Based on promising preliminary results from phase 1 studies (Fitzgerald et al., 2014, 2016) in healthy volunteers (Table 1), the efficacy of inclisiran was tested in a phase 2 double-blind, placebo-controlled RCT in patients with history of CVD or CVD risk equivalents and LDL levels over 70 or 100 mg/dl, respectively (Ray et al., 2017). Patients were treated with the maximum tolerated dose of statins before entering the study and were assigned to receive either a single dose of placebo or inclisiran at doses 200, 300, or 500 mg or two doses of placebo/inclisiran at doses 100, 200, or 300 mg with a 3-month interval. The greatest reduction in LDL-C was detected in the two-dose 300 mg inclisiran group with nearly half of the patients having an LDL cholesterol level below 50 mg/dL 6 months after treatment initiation. Importantly, both PCSK9 and LDL cholesterol levels remained below the baseline across a 8 months follow-up period (Table 2). The most common adverse events (occurring in >2% of patients) included myalgia, headache, fatigue, nasopharyngitis, back pain, hypertension, diarrhea, and dizziness occurred among 11% of inclisiran treated patients compared to 8% receiving placebo. Two deaths were reported, one in a patient with CVD history assigned to the single-dose 500-mg inclisiran group who experienced cardiac arrest and the second in the single-dose 200-mg inclisiran group who died from sepsis. Of note, injection site reaction and transaminasemia were uncommon in inclisiran treated patients, in contrast to high rates of these adverse events reported in studies of ASOs described above.

Ongoing clinical trials are currently assessing the safety, efficacy and tolerability of inclisiran under various different clinical conditions. ORION-2 (NCT02963311) is a phase 2 open-label clinical trial in patients with homozygous FH and ORION-3 (NCT03060577) a phase 2, open-label, non-randomized, extension trial designed to compare inclisiran to evolocumab in high CVD risk patients (history of atherosclerotic CVD, symptomatic atherosclerosis, type 2 DM or FH) with elevated LDL cholesterol levels (Table 4).

#### SPC5001

Inhibition of PCSK9 was also assessed in a phase 1 double-blind, placebo-controlled clinical trial using a 14-mer oligonucleotide with locked nucleic acid (LNA) modifications, SPC5001 (van Poelgeest et al., 2015). In total, twenty-three adult volunteers with mild hypercholesterolemia (fasting LDL-C≥100mg/dl) were enrolled, of whom 17 were exposed to Apo-B SNALP (Apo-B siNA in an LNP formulation) and the rest to placebo. In this first in-human study, SPC5001 reducedPCSK-9 by 49%. Regarding lipid parameters, SPC5001 decreased LDL-C at the end of therapy, but this effect was attenuated 3 weeks post treatment. Importantly, dose-dependent injection site reactions developed in 44% of the SPC5001-treated subjects and transient serum creatinine increases of ≥20 μmol/L (15%) were observed. Four out of six subjects receiving SPC5001 at the highest dose developed renal tubular toxicity and one subject was diagnosed with biopsy-proven acute tubular necrosis resulting in termination of the clinical development of the study drug (Table 1).

### Lipoprotein(a)

Lipoprotein(a) is an independent risk factor for CVD events, especially myocardial infarction (Waldeyer et al., 2017), potentially through accelerated atherosclerosis as a result of intimal deposition and/or prothrombotic or anti-fibrinolytic effect as apolipoprotein (a) possesses structural homology with plasminogen and plasmin. In a large prospective study investigating the association between Lp(a) excess and incident CHD reported an odds ratio of 1.60 (95% CI 1.38–1.85) between the upper and lower thirds of baseline Lp(a) levels after adjustment for traditional cardiovascular risk factors (Bennet et al., 2008). Despite convincing data linking Lp(a) with CVD, there is no definite clinical trial evaluating the effect of lowering Lp(a) on prevention of CHD. Currently, plasma Lp(a) measurement, while not recommended for risk screening in the general population, should be considered in people with high CVD risk or a strong family history of premature atherothrombotic disease (Nordestgaard et al., 2010).

An ASO targeting Lp(a) has been developed and tried in a dose escalating phase 1 study conducted in healthy volunteers with baseline levels of Lp(a) >250 nmol/L (100 mg/dL) (Table 1). Patients assigned to treatment with ISIS-APO(a)_Rx_,received single or multiple (six) subcutaneous injections ranging from 100 to 300 mg over 4 weeks. No decrease in Lp(a) concentration was observed in the single dose group, whereas patients receiving the multiple dose scheme experienced a dose-dependent reduction in Lp(a) levels (−39.6% in the 100 mg group, −59% in the 200 mg group, and −77.8% in the 300 mg group). The most common adverse event was site injection reaction leading to treatment discontinuation in one participant (Tsimikas et al., 2015). Two randomized, placebo-controlled, dose ranging clinical trials were subsequently conducted in order to investigate the efficacy, safety, and tolerability of two unique ASOs -IONIS-APO(a)-LRx and IONIS-APO(a)Rx- designed to lower Lp(a) concentrations (Tables 1, 2). Significant reductions of Lp(a) between 62.8 and 84.5%were observed in the intervention arm compared to the placebo group in both clinical trials. Concerning safety, both ASOs were well-tolerated, although two episodes of myocardial infarction were reported in the IONIS-APO(a)Rx phase 2 trial that were deemed unrelated to the study drug (Viney et al., 2016).

### Angiopoetin-like protein-3

Angiopoetin-like protein-3 has been established as a central regulator of lipoprotein metabolism and loss-of-function variants have been associated with increased insulin sensitivity, reduced free fatty acid circulating levels and decreased plasma lipid levels. A study examining the relationship between ANGPTL3 loss-of-function variants and coronary artery disease in 58,355 adults reported that the presence of an ANGPTL3 loss-of-function variant was associated with a 41% lower odds of CAD (OR 0.59; 95% CI 0.41–0.85) (Dewey et al., 2016). In a phase 1 RCT fourty-four volunteers (with triglyceride levels of either 90–150 mg per deciliter or >150 mg per deciliter, depending on the dose group) were randomly assigned to receive subcutaneous injections of placebo or an ASO targeting *ANGPTL3* mRNA in a single- or multiple doses scheme. Participants receiving the ASO, presented dose-dependent reduction in ANGPT3 levels and both lipids and lipoproteins. In specific, 6 weeks post-treatment, the multiple-dose group presented reduced levels in reduced ANGPTL3 protein (reduction of 46.6–84.5%) triglyceride levels (reduction of 33.2–63.1%), LDL cholesterol (reduction between 1.3 and 32.9%), apolipoprotein B (reduction of 3.4–25.7%) and apolipoprotein C-III (reduction of 18.9–58.8%) and these reductions were significantly higher than those in the placebo group. The agent was well tolerated with no serious adverse events reported, or discontinuations of treatment (Graham et al., 2017; Table 1). Safety and efficacy of ANGPTL3 is now being explored in phase 2 clinical trials in subjects with hypertriglyceridemia, type 2 diabetes mellitus and non-alcoholic fatty liver disease (NCT03371355) and in patients with familial chylomicronemia syndrome (NCT03360747) (Table 4).

## FDA-approved RNA therapeutics

Besides mipomersen, specific ASOs have received FDA approval for use in non-cardiovascular diseases (Table 5). **Fomiversen** [Vitravene, Novartis] is the first ASO to be approved for clinical use in 1998 indicated for cytomegalovirus (CMV) retinitis. This 21-mer phosphorothioate oligodeoxynucleotide targets the mRNA encoding the CMV immediate-early (IE)-2 protein, which is required for viral replication (Vitravene Study Group, 2002). **Pegaptanib** [Macugen, OSI pharmaceuticals, Pfizer] is an aptamer targeting vascular endothelial growth factor (VEGF165) and was approved by the FDA for the treatment of AMD of the retina. This is the leading cause of blindness in people older than 50 years of age and is attributed to VEGF165-stimulated neovascularization of the choroid (Gragoudas et al., 2004). Recently, approval was granted for **eteplirsen** [Exondys 51, Sarepta Therapeutics] to be used in Duchenne muscular dystrophy, a fatal neuromuscular disorder characterized by a mutation in the dystrophin gene. Eteplirsen exerts its action by restoring the translational reading frame of dystrophin mRNA through specific skipping of exon 51 in the defective gene variants, thus promoting dystrophin production (Mendell et al., 2013). Another FDA-approved ASO is **nusinersen** [Spinraza, Biogen] a 18-mer phosphorothioate 2′-O-methoxyethoxy antisense oligonucleotide which modulates alternative splicing of the survival motor neuron (SMN) gene and is indicated for spinal muscular atrophy (Finkel et al., 2017).

**Table 5:** FDA-approved oligonucleotide therapies.

**Brand name [Generic name]**	**Type of treatment**	**Target**	**Disease**	**Year of approval**	**Relevant studies**
Vitravene [Fomivirsen]	ASO	mRNA encoding IE2	CMV retinitis	1998	The Vitravene Study Group, 2002
Macugen, [Pegaptanib]	Aptamer	VEGF165	AMD of the retina	2004	Gragoudas et al., 2004
Kynamro [Mipomersen]	ASO	ApoB-100 mRNA	Homozygous familial hypercholesterolemia	2013	Raal et al., 2010; McGowan et al., 2012; Stein et al., 2012; Thomas et al., 2013
Exondys 51 [Eteplirsen]	SSO	DMD 001-gene (exon 51 target site)	Duchenne muscular dystrophy	2016	Mendell et al., 2013
Spinraza [Nusinersen]	ASO	SMN2 mRNA	Type 1, 2, and 3 spinal muscular atrophy	2016	Finkel et al., 2017

*ASO, antisense oligonucelotides; IE2, immediate early region 2; CMV, cytomegalovirus; VEGF, vascular endothelial growth factor; AMD, age-related macular degeneration; ApoB-100, apolipoprotein B-100; SSO, splice-switching oligo; DMD, Duchenne muscular dystrophy; SMN, survival motor neuron*.

## Conclusion and future perspectives

In conclusion, we are witnessing tremendous advances in RNA therapeutics field and a rapid translation of experimental studies to human clinical trials paving the way toward precision medicine. There are challenges though to be overcome before RNA-based therapeutic agents could efficiently evolve into drugs. A number of chemical modifications have been introduced to enhance target binding affinity, cellular uptake, pharmacokinetics and drug potency along with the development of natural or synthetic carriers to achieve efficient *in vivo* delivery (Wei et al., 2017; Yin et al., 2017). Minimising off-target effects and immunogenicity remains the most challenging setback and significant efforts are being made in order to mitigate unwanted toxicity, before this exciting novel technology could be largely implemented in clinical practice.

To this end, a sophisticated genome editing tool consisting of RNA-guided DNA endonucleases such as Cas9 and CRISPR (clustered regularly interspaced short palindromic repeats) was recently introduced to the scientific community. This versatile tool, in contrast to its “predecessors” RNAi/genome delivery systems, allows suppression (knock out) and/or overexpression (knock in) of a target's expression by introducing a double-stranded break on the site of interest within the genome, which is guided upon RNA oligonucleotides, of 20–21 nt length, complementary to genomic regions of the targeted segment (Cong et al., 2013; Mali et al., 2013). Given that the cellular machinery relies on two known mechanisms by which repairs double-stranded nicks; the non-homologous end joining, which bridges the two newly formed ends, and the homology-directed repair, which utilizes a neighboring template to replace the affected area through homologous recombination; the latter may be exploited in order to introduce site-specific mutations in the genome, supporting further the versatility of this method. Importantly, this system empowers the simultaneous targeting of multiple sites by simply providing more than one different RNA guides that are directed toward different genomic regions later subjected to Cas9 “nicking.” Since the endonuclease, Cas9, is not endogenously expressed in human or mice systems, but only in bacteria, like Streptococcus pyogenes (Sp) from which has been originally isolated (Sapranauskas et al., 2011; Jinek et al., 2012), a forced expression of Cas9 to the studied system is a prerequisite that also features a common laboratory practice hurdle when it comes to transfection-resistant systems, e.g primary cells and thus the delivery strategy of Cas9 shall be extensively considered in advance and tailored accordingly. This innovative technology has already been employed to “correct” disease contexts like Duchenne muscular dystrophy (Long et al., 2016) hereditary tyrosynemia type I (Yin et al., 2016) and lethal metabolic liver disease (Yang Y. et al., 2016) in animal models. However, despite these promising outcomes there are several limitations regarding the CRISPR–Cas9 system that have to be acknowledged: (a) off-target activity resulting in unwanted mutations, (b) low efficiency of genome editing using homology-directed repair (HDR) and (c) challenging delivery of CRISPR–Cas9 components into desired tissues using both viral and non-viral methods (Long et al., 2016; Tycko et al., 2016; Komor et al., 2017). Of particular interest, similar strategies have been developed for rendering feasible the editing of the transcriptome (Abudayyeh et al., 2016) instead of the genome. We, and others, have previously underpinned the importance of adenosine (A)-to-inosine (I) RNA editing, a widespread RNA modification (Stellos et al., 2016) in human transcriptome, in RNA metabolism thus modulating the context of several diseases (Choudhury et al., 2012; Chen et al., 2013; Yamashita et al., 2013; Shoshan et al., 2015) including atherosclerotic heart disease (Stellos et al., 2016), as we have rigorously reviewed (Gatsiou et al., 2017). Recently, a breakthrough proof-of-principle study, documented that the use of Cas system in conjunction with the catalytic activity of an A-to-I RNA editing enzyme, namely ADAR2, is able to edit and correct disease-relevant mutations of full-length transcripts, previously introduced into immortalized human cells (Cox et al., 2017). In a different approach, another group fused the catalytic domain of ADAR2 with an RNA binding domain directed to a specific stem loop sequence embedded within the RNA guide, restoring in this way the function of a neurodevelopmental disorder-associated protein in primary neurons (Sinnamon et al., 2017). Whether these concepts can be adopted first in animal models at a preclinical stage manipulating endogenous A-to-I RNA editing within specific transcripts, of which A-to-I RNA editing levels have been previously linked with a particular disease context, in order to attenuate the progression of a disease remains yet to be investigated. Nevertheless, these “trail blazer” findings provide convincing argumentation prompting us to contemplate that genome and mainly transcriptome editing feature as dynamic new additions to the “quiver” of RNA therapeutics landmarking the dawn of a new era in RNA therapeutics (Stellos, 2017a).

Without doubt, we are heading toward the new era of precision medicine based on the discipline of an evidence-based approach customized to patient-specific unique characteristics (MacRae et al., 2016). Precision medicine is gaining ground over the traditional “one-size-fits-all” medical treatment approach (Houser, 2016; MacRae and Seidman, 2017) by enabling the identification of the exact underlying molecular mechanism of the disease and the design of therapeutic interventions deployed specifically for this mechanism (Benjamin et al., 2017). A bold, innovative research effort is now emerging based on the fundamental concept of individualized treatment taking into account individual variability in genes, environment and lifestyle (PMI Working Group report to the Advisory Committee to the Director, NIH, 2015^1^) in order to identify which prevention strategy and treatment is effective in each patient (MacRae et al., 2016; Shah et al., 2016). Advances in precision medicine will soon be applicable in cardiology and medicine.

## Author contributions

KoS initiated this review study, designed its structure, provided conceptual advice to all coauthors and revised critical parts of it. AL wrote the first draft of the manuscript. AG provided conceptual advice on the basic science part of the manuscript and wrote the future perspectives part and revised the whole manuscript. GG organized data collected from clinical trials into tables and revised the manuscript. KiS provided conceptual advice on the organization of the information into the tables. All authors contributed to manuscript revision, read and approved the submitted version.

### Conflict of interest statement

The authors declare that the research was conducted in the absence of any commercial or financial relationships that could be construed as a potential conflict of interest.

## Footnotes

^1^http://www.nih.gov/precisionmedicine/09172015-pmi-working-group-report.pdf
